# *Cleistopholis patens* root bark extract exerts cardioprotective effect against doxorubicin-induced myocardial toxicity in rats

**DOI:** 10.1186/s42826-024-00225-3

**Published:** 2024-11-18

**Authors:** Chidinma Pamela Ononiwu, Parker Elijah Joshua, Christian Chijioke Amah, Rita Onyekachukwu Asomadu, Ekezie Matthew Okorigwe, Chukwubuikem Stephen Nnemolisa, Timothy Prince Chidike Ezeorba, Valentine Odirachukwumma Nwanelo, Favour Chinagorom Iyidiegwu, Justin Onuawuchi Duru, Peace Nkiruka Okeke, Onyinyechi Becky Adiele

**Affiliations:** 1 Department of Biochemistry, Faculty of Biological Sciences, University of Nigeria, Nsukka, 410001 Enugu State Nigeria; 2 Department of Biochemistry, Faculty of Natural and Applied Sciences, State University of Medical and Applied Sciences Igbo-Eno, Nsukka, Enugu State Nigeria; 3 Department of Molecular Biotechnology, School of Biosciences, University of Birmingham, Edgbaston, B15 2TT UK; 4 Department of Biotechnology, School of Medicine, 3900 University Blvd., Tyler, TX 75799 USA; 5 Department of Science Laboratory Technology, Faculty of Physical Sciences, University of Nigeria, Nsukka, 410001 Enugu State Nigeria

**Keywords:** Myocardial infarction, Doxorubicin, *Cleistopholis patens*, Cardiotoxicity, Oxidative stress, Histopathology

## Abstract

**Background:**

Myocardial Infarction still persists as the most prevalent cardiovascular disease and is a top cause of morbidity and mortality in doxorubicin treated cancer patients. This study evaluated the prophylactic effect of the ethanol root bark extract of *Cleistopholis patens* (ERBECP) against doxorubicin-induced myocardial infarction in wistar rats. Extraction, preliminary phytochemical analysis, acute toxicity study and body weight (b.w.) of ERBECP were achieved using standard methods. Phyto-constituents in ERBECP were indentified using Gas Chromatography-Mass Spectrometry (GC–MS) technique. Thirty (30) male albino Wistar rats of average b.w. ranging between 100 and 130 g were divided into six groups of five rats each. Groups I, II and III served as normal, doxorubicin (DOX) and standard (Vasoprin 150 mg/kg b.w) controls respectively, while groups IV, V and VI were orally pre-treated with the extract (200, 400 and 600 mg/kgb.w) for two weeks prior to intraperitoneal induction of cardiotoxicity with DOX (20 mg/kg bw) on day 14.

**Results:**

Disturbances in serum cardiac function bio-markers such as; Cardiac Troponin-I (CTnI), Creatine Kinase (CK), Lactate Dehydrogenase (LDH), Aspartate aminotransferase (AST), Alanine aminotransferase (ALT). Lipid profile markers such as; Total cholesterol (TC), Triacylglycerol (TAG), Low Density Lipoprotein (LDL), High Density Lipoprotein (HDL). Oxidative stress markers such as; Malondialdehyde (MDA), Superoxide Dismutase (SOD), Catalase (CAT), Glutathione (GSH) confirmed the induction of myocardial infarction. Histological assessment of heart tissues was performed to validate biochemical results. The GC–MS analysis of ERBECP identified a total of 69 compounds. Safety profile of the aqueous extract was safe for the animals up to the highest dose of 5000 mg/kg b.w. Pre-treatment of DOX group with ERBECP could significantly increase the b.w. compared to the DOX-treated group during the experimental period of 2 weeks. There were significant (*p* < 0.05) alterations in the levels of CTnI, CK, LDH, AST, ALT and lipid profile indices in the DOX control rats. Also, significant (*p* < 0.05) increase was observed in MDA and decrease in SOD, CAT and GSH in the DOX control rats. However, administration of the extract significantly (*p* < 0.05) normalized these alterations and reversed the architectural changes in the heart. The 69 compounds were screened against the target protein (CBR1); we identified seven hits based on the docking score and interactions with the active site residues. All the *C. patens* constituents had MW (g/mol) less than 500, HBA < 10 and HBD not more than 5. Apart, 9-Octadecenoic acid (Z)-, 2,3-dihydroxy propyl ester and Estra-1,3,5(10)-trien-17. beta. -ol, all the constituents had LD_50_ lower than 2000 mg/kg.

**Conclusions:**

The findings reveals ERBECP demonstrated promising potential and can be exploited in the development novel cardiac therapeutic agents.

## Background

Myocardial Infarction represents one of the prevalent causes of morbidity and mortality in industrialized nations [1]. The loss of cardiac function due to prolonged oxygen deprivation coupled with the poor regenerative capacity of the adult human heart account for about 16.7 million deaths annually [1–4]. Myocardial necrosis following a complete loss of blood flow to the cardiomyocytes for more than 20 min is inevitable and conditions such as atherosclerosis of the coronary arteries may lead to an ischemic myocardium [5].

The clinical usage of doxorubicin (DOX) which is a secondary metabolite of *Streptomyces peucetius* var. caesius and an anthracycline antibiotic known for its highly effective anti-neoplastic potential has been restricted due to its dose-dependent cardiotoxic side effects [6–8]. This DOX- induced cardiac complication may occur early or late during the course of treatment or it may appear months or even years after cessation of chemotherapy [9]. Cardiomyopathy, electrocardiograhic (ECG) changes, cardiac arrhythmia and acute heart failure are some of the manifestations of cardiac damage caused by DOX[10]. Molecular processes such as intracellular calcium dysregulation, iron metabolism, sarcomere alterations, gene expression modulation, apoptosis and oxidative stress play key roles in the pathophysiology of DOX- induced cardiac damage [11]. The loss of myocardial mitochondria function due to the generation of reactive oxygen species (ROS) has been proposed to be commonly responsible for DOX- induced cardiotoxicity and the heart is extremely predisposed to this oxidative damage partly due to the presence of minimal levels of antioxidant enzymes [8, 11]. The redox cycle prompted when the semiquinone metabolite of DOX reacts with molecular oxygen, in addition to the disruption of energetic metabolism upon the binding of DOX to cardiolipin displacing the cardiolipin-electron transport protein interface in the mitochondrial inner membrane result in the formation of superoxide anion radicals [6, 8]. Nitric oxide synthases (NOS) and nicotinamide adenine dinucleotide phosphate (NADPH) oxidases pathways represent other sources of DOX-induced ROS production in the myocardium. These enzymes interact with DOX to trigger oxidative stress [11]. The development of novel therapeutic strategies is pertinent in counteracting the cardiovascular side effects of DOX and presently, natural products derived from medicinal plants are at the forefront of disease prevention and treatment [12, 13].

*Cleistopholis patens* (Family: Annonaceae) is a sun-loving tree, with a height of about 20–30 m. It is a fast-growing plant, frequently found in Africa. *C. patens* is commonly referred to as ‘salt and oil tree’ because the sap is reddish, looking like palm oil [14]. Nigeria and other African countries, employ the root bark and leaves in the treatment of several diseases such as malaria, urogenital and other bacterial infections [15]. Also, alkaloids and 3-methoxy champangine from the ethanol extract of *C. patens* have been identified as potent anti-fungal agents [15]. The insecticidal strength of *C. patens* against *Sitotroga cerealella* infestation in rice grains in Nigeria have been reported [16]. Studies also reveal that the usage of the root bark powder of *C. patens*on humans will not cause any deleterious effects since it is non-toxic to albino rats [14, 17]. It is used in folklore medicine to treat heart related maladies in the South- Eastern part of Nigeria and shows very strong hypolipidemic activity [18, 19].

Centered on the traditional exploitation of *C. patens* in ameliorating cardiac complications, this study was designed to scientifically validate its usage and to investigate the cardioprotective effects of its ethanolic root bark extract on DOX -induced myocardial infarction in experimental animals. Biochemical serum cardiac function markers, lipid profile, antioxidant and lipid peroxidation biomarkers levels were determined along with histopathological assessment of the heart tissues.

## Methods

### Plant materials

Fresh root barks of *C.* patens were procured from a habitat in Agu-Orba in Udenu Local Government Area of Enugu State, Nigeria. The root barks were identified and authenticated by Mr. Alfred Ozioko, a taxonomist with the International Center for Economic and Drug Development (InterCEDD) Research Centre, Nsukka, Enugu State. Plant sample was also deposited (Voucher No. InterCEDD/505) at the herbarium for reference purposes.

### Experimental animals

Thirty (30) adult male albino Wistar rats of average b.w. ranging between 100 and 130 g were employed for this study. They were purchased from the Animal Breeding Unit, Faculty of Veterinary Medicine, University of Nigeria, Nsukka. The rats were acclimatized under standard laboratory condition with 12-h light/dark cycle in the Animal House of the Faculty of Veterinary Medicine for one week prior to the commencement of the experiment. They were maintained on a regular feed and portable water ad libitum. They received humane care throughout the experimental period in accordance with the institutional Ethical Clearance (EC approval number, UNN/ FBS/EC/1075), recommendations for care and use of laboratory animals.

### Chemicals and reagents

All chemicals used in this study were of analytical grade. They were products of Sigma Aldrich, St. Louis, MO (standard phytochemicals, ammonia, sodium hydroxide, ethyl acetate, acetic anhydride, ferric chloride, and formaldehyde), British Drug House (BDH), England (methanol, ethanol, hydrochloric acid, and sulphuric acid), Qualikems, India (citric acid, aluminium chloride, and phosphomolybdic acid), Fluka, Germany (lead acetate and potassium ferrocyanide) and May and Baker, England (sodium chloride, dextrose, and sodium citrate). Biochemical assay kits were products of Randox and Roche Diagnostics.

### Plant preparation

The fresh root barks of *C. patens* were collected and washed to remove impurities. These root barks were then shade-dried with intermittent turning to prevent decay, until crispy. The dried root bark was pulverized into powdered form using a mechanical grinder. 3000 g of the pulverized root bark was macerated in 10 L absolute ethanol using a maceration flask. The mixture stood for 72 h with occasional stirring; it was filtered into a flat-bottomed flask using a muslin cloth. Further filtration was achieved with Whatman No. 1 filter paper in order to eliminate fine residues. The filtrate was concentrated in-vacuum to obtain the crude ethanol extract. The concentrated extract was stored in a labeled sterile screw-capped bottle at 2–8 °C.

### Preliminary phytochemical analysis of plant extract

The preliminary qualitative and quantitative phytochemical analyses of the ethanol root bark extract of *C. patens* were carried out to ascertain the presence of biologically active compounds. The determinations were done using standard protocols as illustrated by [20, 21].

### Gas chromatography-mass spectrometry (GC–MS) analysis

Using GC–MS equipment (Thermo Scientific Co.), Thermo GC-TRACE ultra ver.: 5.0, Thermo MS DSQ II, the phytochemical analysis of the ethanol extract was carried out. The GC–MS system was used in the following experimental conditions: TR 5-MS capillary standard non-polar column, measures 30 m, has an ID of 0.25 mm, and a film thickness of 0.25 μm. Mobile phase (carrier gas: He) flow rate was calibrated at 1.0 ml/min. In the gas chromatography section, the injection volume was 1 μl and the temperature was programmed (oven temperature) to be 40 °C rising to 250 °C at 5 °C/min. The data were compared using the Wiley Spectral library search tool, with samples dissolved in chloroform run fully at a range of 50–650 m/z.

### Acute toxicity test

The method of Lorke [22] was used to determine the median lethal dose LD_50_ in two phases.

### Induction procedure

The animals were randomly divided into six groups of five rats each. DOX (50 mg) was dissolved in 25 ml of normal saline and was injected intraperitoneally (20 mg/kg b.w) to induce myocardial infarction in groups II, III, IV, V and VI, respectively [23]. This was done on the 14th day after the final doses of standard drug, and extract were administered to the rats. The rats received oral treatment of standard drug, Vasoprin and a graded dose of the ERBECP once daily for two weeks. This was in accordance with the method as described by [24]. The changes of b.w. of rats in different groups were monitored.

### Experimental design

Group I: Control; Group II: Dox 20 mg/kg b.w; Group III: Dox 20 mg/kg b.w + Vasoprin 150 mg/kg b.w; Group IV: Dox 20 mg/kg b.w + ERBECP 200 mg/kg b.w; Group V: Dox 20 mg/kg b.w + ERBECP 400 mg/kg; Group VI: Dox 20 mg/kg b.w + ERBECP 600 mg/kg b.w

### Biochemical investigations

The cardiac disease was confirmed 72 h post induction, after which the rats were euthanized and their blood samples were obtained via ocular puncture and centrifuged to obtain the serum. The obtained serum was subsequently analyzed for cardiac parameters such as CTnI, CK and LDH following manufacturer’s protocols as contained in the enzyme linked-immunosorbent assay (ELISA) kit. AST, ALT and lipid profile parameters such as TC, TAG, HDL and LDL were determined by standard methods as outlined in the RANDOX commercial kit. Antioxidant and lipid peroxidation biomarkers such as MDA, SOD, CAT and GSH were determined by methods of [21–25] respectively.

### Histopathological analysis

Sections of the heart were subjected to histopathological examination. The samples were fixed in 10% phosphate buffered formalin for a minimum of 48 h. The tissues were subsequently trimmed, dehydrated in 4 grades of alcohol (70%, 80%, 90%, and absolute alcohol), cleared in 3 grades of xylene and embedded in molten wax. On solidifying, the blocks were sectioned, 5 µm thick with a rotary microtome, floated in water bath and incubated at 60 °C for 30 min. The 5 µm thick sectioned tissues were subsequently cleared in 3 grades of xylene and rehydrated in 3 grades of alcohol (90%, 80% and 70%). The sections were then stained with Hematoxylin for 15 min. Blueing was done with ammonium chloride. Differentiation was done with 1% acid alcohol before counterstaining with Eosin. Permanent mounts were made on degreased glass slides using a mountant; DPX. The prepared slides were examined with a Motic™ compound light microscope using × 4, × 10 and × 40 objective lenses.

### Protein preparation

DOX is metabolized by a class of cytosolic enzymes called carbonyl reductases. When DOX dosage is sufficiently high, carbonyl reductases convert DOX to doxorubicinol. This alcohol metabolite builds up in cardiac tissues, impairing both systolic and diastolic cardiac function [25]. The 3-dimensional (3D) structure of carbonyl reductase 1(CBR1) with PDB ID: 1WMA was retrieved from the Protein Data Bank (https://www.rcsb.org). Before performing molecular docking, the protein PDB structure was refined using the Protein Preparation Wizard of Schrodinger Suite [26]. Charges and bond order were assigned to the protein and water molecules were deleted to avoid inaccurately high binding scores [26]. Additionally, hydrogen atoms and missing residues were added to the protein. Using the Optimal Potentials for Liquid Simulations (OPLS4) force field, the heavy atom's Root-Mean-Square Deviation (RMSD) was set at 0.30 Å. Finally, we used a neutral pH to optimize amino acids [26]. After creating a receptor glide grid, our hit compounds were molecularly docked to the receptor.

### Ligand preparation

To examine each molecule found by GC–MS analysis, docking studies were conducted to examine any potential interactions with the target protein. The NCBI PubChem database (https://pubchem.ncbi.nlm.nih.gov/) provided the compound structures for download in the structure-data file (SDF) format. To assign correct bond ordering and generate a three-dimensional geometry, ligand preparation was carried out on the downloaded SDF files using the Ligprep with OPLS4 force field [27, 28].

### Receptor grid generation

The prepared protein was used to generate the receptor grid. The co-crystallized ligand was separated from the active site of the prepared protein. The charge cutoff for polarity was 0.25, and the van der Waal radii of the protein atoms were scaled by 1.0. Using the Glide default parameters, a grid centered at the ligand was created. This grid structure was where all ligands were docked.

### Molecular docking

Using the glide implemented in the Schrödinger suites, a molecular docking experiment was carried out. Flexible docking was carried out on a specified receptor grid by utilizing the extra precision (XP) feature of the Glide module. For ligand non-polar atoms, the van der Waals scaling factor was set to 0.15 and 0.85, respectively. There was no stated restriction on the defined ligand-receptor interactions. To see the output of the final docking studies from the pose viewer, the structure output format was set to the pose viewer file. Based on their anticipated interactions and binding affinities with the target, the chosen compounds were ranked.

### Validation of docking method

The method described by [29] was used to validate the docking method. The cognate crystallized ligand was removed from the protein’s binding site. It was then re-docked using the extra precision Glide docking technique. The results were analyzed were analyzed using the hydrogen bonding interactions and the Root Mean Square Deviation (RMSD) between the observed X-ray crystallographic conformation and the expected conformation.

### Drug-likeness and toxicity prediction

The online tool on the SwissADME (https://www.swissadme.ch/index.php) and ProTox-II webserver (https://tox-new.charite.de/protox_II/) were used to make in silico prediction of molecular descriptors, drug-like properties and toxicity profile of *C. patens* constituents. MW (molecular weight), cLogP, HBD (number of hydrogen bond donors), HBA (number of hydrogen bond acceptors), gastro-intestinal absorption, TPSA (Topological polar surface area) and nViolation (number of violations of Lipinski’s rule of five) were calculated based on Lipinski's rule of five.

### Statistical analysis

Data obtained from the laboratory were analyzed using one-way analysis of variance (ANOVA) to compare means across groups in Statistical Product and Service Solution (SPSS), version 20.0. The results were presented as mean ± SD in tables. Mean values with p < 0.05 were considered significant.

## Results

### Phytochemical constituents of the ERBECP

The preliminary phytochemical constituents of the ERBECP revealed the presence of steroids (1.6057 ± 0.0015 mg/100 g), terpenoids (1.4783 ± 0.0006 mg/100 g), phenols (1.2193 ± 0.0208 mg/100 g), flavonoids (1.1790 ± 0.0236 mg/100 g) and alkaloids (1.1597 ± 0.0025 mg/100 g) in high concentrations; glycosides (1.0237 ± 0.0015 mg/100 g) in moderate concentrations and tannins (0.6860 ± 0.0082 mg/100 g) and reducing sugars (0.2060 ± 0.0364 mg/100 g) were detected in low concentrations as shown in Table 1.

**Table 1:** Qualitative and quantitative phytochemical analyses of the ethanol root bark extract of *C. patens*

Phytochemical constituents	Qualitative Analyses	Quantitative Analyses (mg/100 g)
Steroids	+ + +	1.6057 ± 0.0015
Terpenoids	+ + +	1.4783 ± 0.0006
Phenols	+ + +	1.2193 ± 0.0208
Flavonoids	+ +	1.1790 ± 0.0236
Alkaloids	+ +	1.1597 ± 0.0025
Glycosides	+ +	1.0237 ± 0.0015
Tannins	+	0.6860 ± 0.0082
Reducing Sugar	+	0.2060 ± 0.0364

Results are expressed in Means ± SD (n = 3)

+ found to be slightly present in the ERBECP

+ + found to be moderately present in the ERBECP

+ + + found to be highly present in the ERBECP

### Compounds identified in ERBECP using GC–MS analysis

**Fig. 1:**
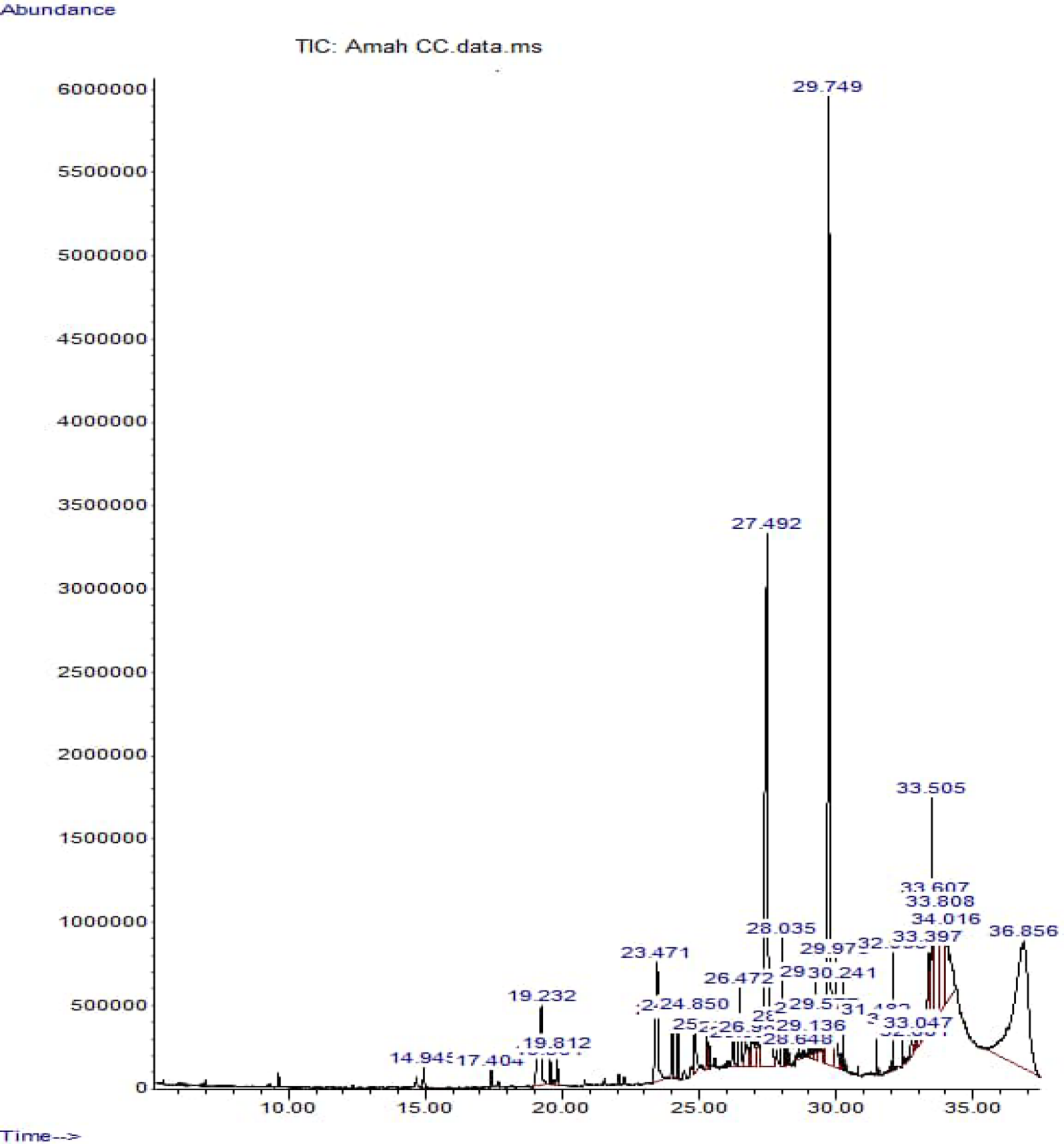
GC–MS Chromatogram of the essential compounds found in ERBECP

The GC–MS analysis of ERBECP identified a total of 69 compounds. While Table 2 showed *C. patens* constituents, their retention time, molecular formula, molecular weight, and peak area, Fig. 1 presented the chromatogram. The chemical constituents identified in the GC–MS analysis of *C. patens* extract are presents in Table 2 and its chromatogram represents in Fig. 1.

**Table 2:** Compounds identified in ERBECP using GC–MS analysis

S/N	Name of compound	RT (min)	Molecular Formula	Molecular Weight (g/mol)	Peak Area (%)	Structures of compound
1	Tetradecane	14.945	C_14_H_30_	198.39	0.26	
2	2,4-Di-tert-butylphenol	17.404	C_14_H_22_O	206.32	0.26	
3	Phenol, 3,5-bis(1,1-dimethylethyl)	17.404			0.26	
4	Dodecanoic acid	19.232	C_12_H_24_O_2_	200.32	2.83	
5	3-Eicosene, (E)-	19.564	C_20_H_40_	280.5	0.31	
6	9-Eicosene, (E)-	19.564			0.31	
7	1-Pentadecene	19.564	C_15_H_30_	210.4	0.31	
8	Hexadecane	19.812	C_16_H_34_	226.44	0.40	
9	Tetradecanoic acid	23.471	C_14_H_28_O_2_	228.37	2.90	
10	5-Eicosene, (E)-	24.002			0.77	
11	1-Docosene	24.002	C_22_H_44_	308.6	0.77	
12	Octadecane	24.214	C_18_H_38_	254.5	0.84	
13	Heptadecane, 2-methyl-	24.214	C_18_H_38_	254.5	0.84	
14	Docosyl propyl ether	24.850	C_25_H_52_O	368.7	1.09	
15	Propyl tetracosyl ether	24.850	C_27_H_56_O	396.7	1.09	
16	Butyl octacosyl ether	24.850	C_32_H_66_O	466.9	1.09	
17	Heptylcyclohexane	25.287	C_13_H_26_	182.35	0.45	
18	Cyclohexane, (4-methylpentyl)-	25.287	C_12_H_24_	168.32	0.45	
19	Cyclohexane, butyl-	25.287	C_10_H_20_	140.27	0.45	
20	9-Heptadecanone	25.351	C_17_H_34_O	254.5	0.43	
21	Cycloundecanol, 1-methyl-	25.351	C_12_H_24_O	184.32	0.43	
22	Carbonic acid, prop-1-en-2-yl tetradecyl ester	26.259	C_18_H_34_O_3_	298.5	0.56	
23	Carbonic acid, prop-1-en-2-yl tridecyl ester	26.259	C_17_H_32_O_3_	284.4	0.56	
24	Pentadecanoic acid, 14-methyl-, methyl ester	26.472	C_17_H_34_O_2_	270.5	1.63	
25	Hexadecanoic acid, methyl ester	26.472	C_17_H_34_O_2_	270.5	1.63	
26	Dodecane, 1,2-dibromo-	26.694	C_12_H_24_Br_2_	328.13	0.85	
27	Isobutyl tetratriacontyl ether	26.694	C_38_H_78_O	551	0.85	
28	Z-8-Hexadecene	26.985	C_16_H_32_	224.42	1.14	
29	Eicosyl propyl ether	26.985	C_23_H_48_O	340.6	1.14	
30	Carbonic acid, heptadecyl isobutyl ester	26.985	C_22_H_44_O_3_	356.6	1.14	
31	n-Hexadecanoic acid	27.492	C_16_H_32_O_2_	256.42	0.85	
32	1-Octadecene	28.035	C_18_H_36_	252.5	1.63	
33	Cycloeicosane	28.035	C_20_H_40_	280.5	1.63	
34	Methoxyacetic acid, 2-tetradecyl ester	28.205	C_17_H_34_O_3_	286.4	0.44	
35	Nonadecane, 2-methyl-	28.205	C_20_H_42_	282.5	0.44	
36	Undecane, 2,10-dimethyl-	28.205	C_13_H_28_	184.36	0.44	
37	Estra-1,3,5(10)-trien-17.beta.-ol	28.648	C_18_H_24_O	256.399	0.18	
38	Pentadecafluorooctanoic acid, octadecyl ester	28.648	C_26_H_37_F_15_O_2_	666.5	0.18	
39	17-Pentatriacontene	28.648	C_35_H_70_	490.9	0.18	
40	Cyclohexane, pentyl-	29.039	C_11_H_22_	154.29	0.38	
41	Hexane, 1,6-dicyclohexyl-	29.039	C_18_H_34_	250.5	0.38	
42	Heptafluorobutyric acid, hexadecyl ester	29.136	C_20_H_33_F_7_O_2_	438.5	0.31	
43	Heptadecafluorononanoic acid, hexadecyl ester	29.136			0.31	
44	Octacosanol	29.136			0.31	
45	9-Octadecenoic acid (Z)-, methyl ester	29.251			0.75	
46	cis-13-Octadecenoic acid, methyl ester	29.251			0.75	
47	trans-13-Octadecenoic acid, methyl ester	29.251			0.75	
48	Methyl stearate	29.577			0.59	
49	cis-Vaccenic acid	29.749			18.26	
50	9-Octadecenoic acid, (E)-	29.749			18.26	
51	cis-13-Octadecenoic acid	29.749			18.26	
52	Octadecanoic acid	29.973			1.62	
53	1-Eicosene	30.241			0.52	
54	Cyclohexadecane, 1,2-diethyl-	30.241			0.52	
55	Heptadecyl trifluoroacetate	31.482			0.27	
56	Bis(2-ethylhexyl) phthalate	32.063			0.63	
57	Diisooctyl phthalate	32.063			0.63	
58	1-Hexacosene	32.437			0.16	
59	9-Octadecenoic acid (Z)-, 2,3-dihydroxypropyl ester	32.750			0.30	
60	Oleic Acid	32.750			0.30	
61	Oxirane, tetradecyl-	32.881			0.17	
62	13-Octadecenal, (Z)-	32.881			0.17	
63	cis-11-Hexadecenal	32.881			0.17	
64	2- Chloropropionic acid, hexadecyl ester	33.397			2.36	
65	Squalene	33.505			3.14	
66	6,11-Dimethyl-2,6,10-dodecatrien-1-ol	33.505			3.14	
67	9-Octadecenoic acid (Z)-, 2-hydroxy-1-(hydroxymethyl)ethyl ester	33.607			7.01	
68	Oxirane, tridecyl-	33.607			7.01	
69	9-Tetradecenal, (Z)-	36.856			19.65	
	RT = Retention Time

### Acute toxicity profile of the ERBECP

The result on Table 3 of acute toxicity test showed that ERBECP was not lethal even after extract administration at the highest dosage (5000 mg/kg b.w.). There was no significant change in the body weight and behavior of mice after 24 h.

**Table 3:** The phase I and II of the acute toxicity study on ERBECP

Phases/Groups	Dosage of extract (mg/kg b.w)	Mortality rate
*Phase I*		
Group 1	10	0/3
Group 2	100	0/3
Group 3	1000	0/3
*Phase II*		
Group 1	1600	0/3
Group 2	2900	0/3
Group 3	5000	0/3

### Effect of the ERBECP on the body weight changes of rat induced-DOX

**Fig. 2:**
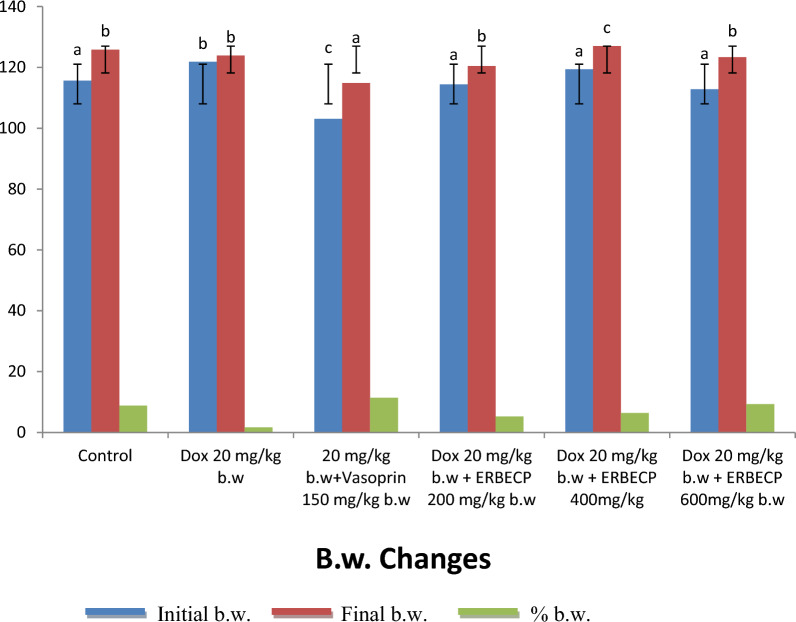
Effect of ERBECP on the body weight changes in rats induced with myocardial infarction. Mean values with different letters as superscripts across the groups are considered significantly different at p < 0.05, while mean values with the same letters as superscripts across the groups are considered non-significant at p > 0.05

Changes in b.w. of rats treated with ethyl ERBECP and standard drug (Fig. 2). The results showed a significant (*p* < 0.05) increases in final b.w. of rats in comparison to the values obtained for the initial b.w. rats. It was demonstrated that Dox 20 mg/kg b.w. treatment resulted in reduced body weights compared with the control group at % 1.68. Meanwhile, the groups that received Dox 20 mg/kg b.w + ERBECP 600 mg/kg b.w and 20 mg/kg b.w + Vasoprin 150 mg/kg b.w had a % b.w. of 9.3 and 11.43 respectively which are comparable to the control.

### Effect of the ERBECP on cardiac biomarkers

**Fig. 3:**
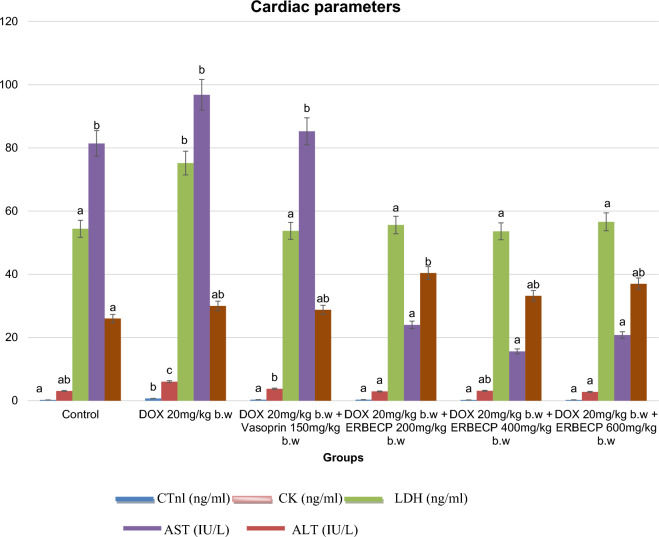
Effect of ERBECP on the cardiac parameters in rats induced with myocardial infarction. Mean values with different letters as superscripts across the groups are considered significantly different at p < 0.05, while mean values with the same letters as superscripts across the groups are considered non-significant at p > 0.05

The DOX control rats exhibited a significant increase (*p* < 0.05) in CTnI, CK, LDH, AST and ALT in contrast to those of the normal control (Fig. 3). However, administration of ERBECP notably decreased (*p* < 0.05) the serum levels of these indices except for ALT serum levels that remained non-significantly higher (*p* > 0.05) despite treatment with the root bark extract.

### Effect of the ERBECP on lipid profile biomarkers

**Fig. 4:**
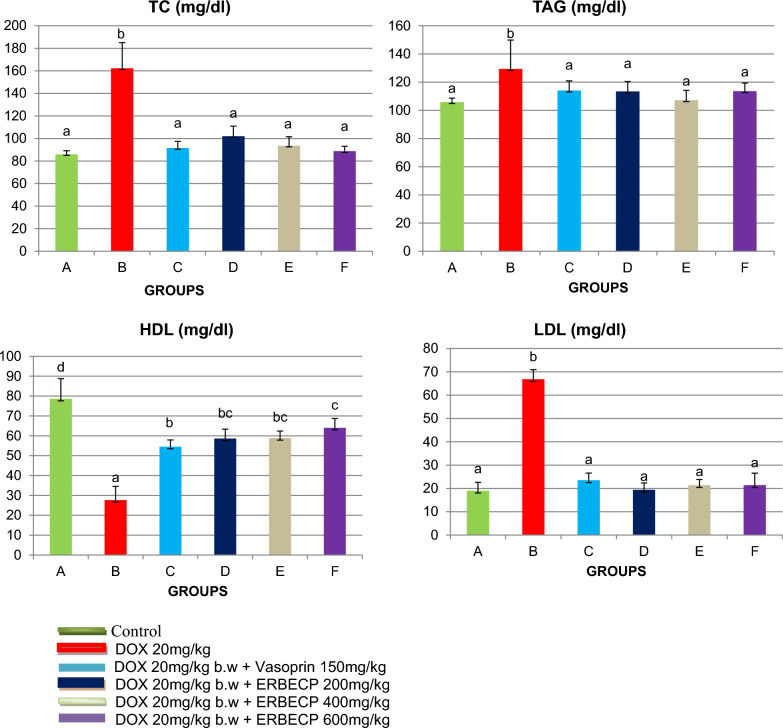
Effect of ERBECP on the lipid profile parameters in rats induced with myocardial infarction. Mean values with different letters as superscripts across the groups are considered significantly different at p < 0.05, while mean values with the same letters as superscripts across the groups are considered non-significant at p > 0.05

The concentrations of TC, TAG and LDL were significantly elevated (*p* < 0.05) and that of HDL significantly reduced (*p* < 0.05) in the DOX control rats compared to the normal control (Fig. 4). However, there was observed restoration of these alterations in the treated groups.

### Effect of the ERBECP on MDA and antioxidant biomarkers

**Fig. 5:**
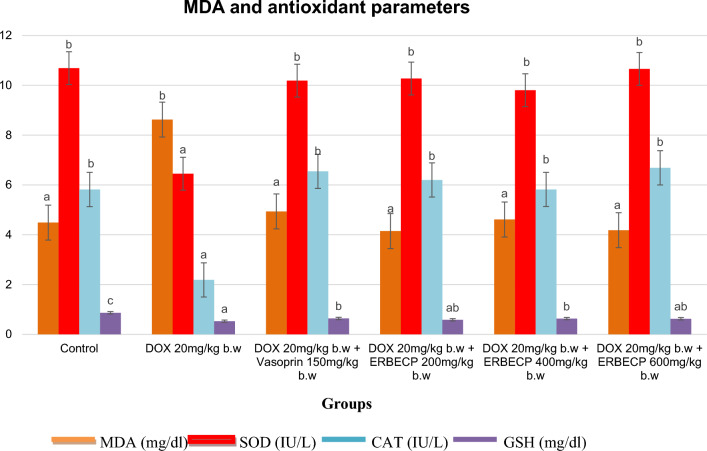
Effect of ERBECP on the MDA and antioxidant parameters in rats induced with myocardial infarction. Mean values with different letters as superscripts across the groups are considered significantly different at p < 0.05, while mean values with the same letters as superscripts across the groups are considered non-significant at p > 0.05

The Fig. 5 showed that DOX control rats demonstrated a significant decrease (*p* < 0.05) in the activities of SOD and CAT as well as a significant decrease (*p* < 0.05) in GSH levels in comparison to group 1 rats. MDA levels were found to be significantly higher (*p* < 0.05) in group 2 rats in contrast to the normal control rats. Treatment with extract significantly (*p* < 0.05) reduced and increased MDA and GSH levels respectively. Also, SOD and CAT activities were greatly improved in the treated groups.

### Histopathology of the heart tissues

**Fig. 6:**
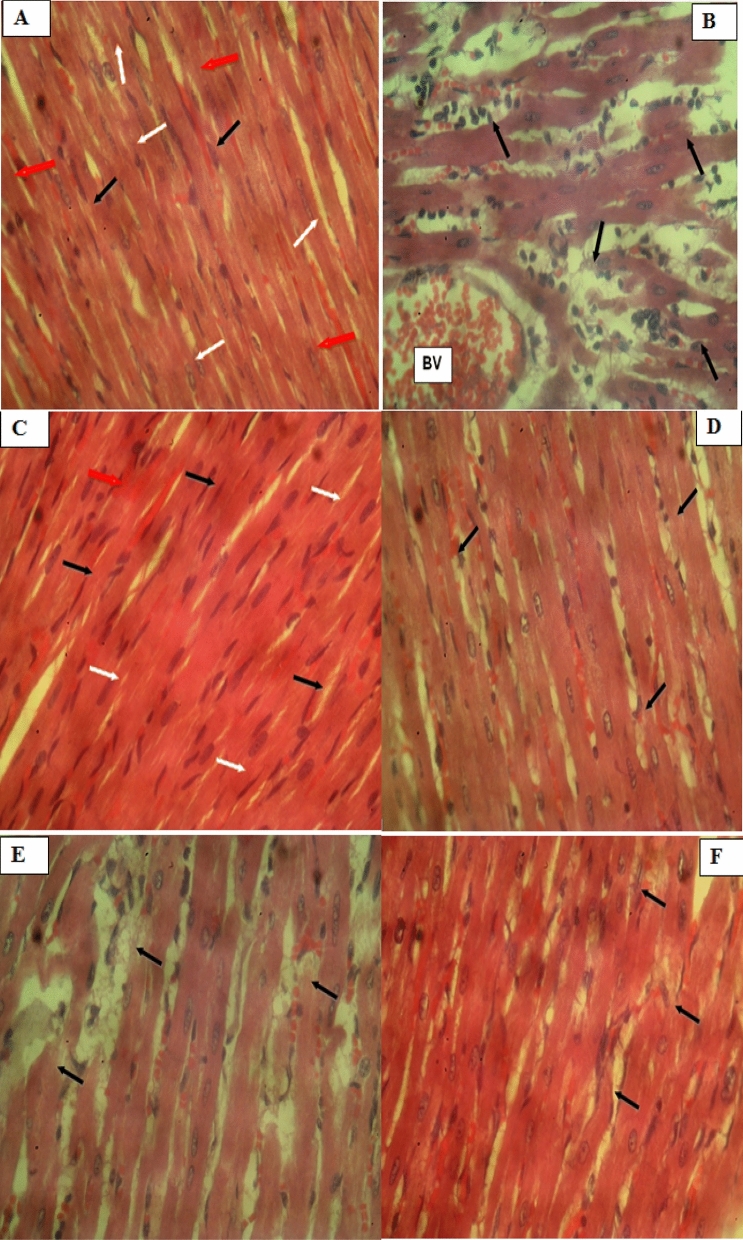
Photomicrograph of heart tissues of experimental rats. (**a**) heart tissue showing normal myocardial histomorphology(H&Ex400). (**b**) heart tissue showing myocardial degeneration and necrosis (H&Ex400). (**c**) heart tissue showing normal myocardial histomorphology (H&Ex400). (**d**) heart tissue showing mild myocardial degeneration (H&Ex400). (**e**) heart tissue showing moderate myocardial degeneration (H&Ex400). (**f**) heart tissue showing relatively normal myocardial degeneration (H&Ex400)

**Fig. 7:**
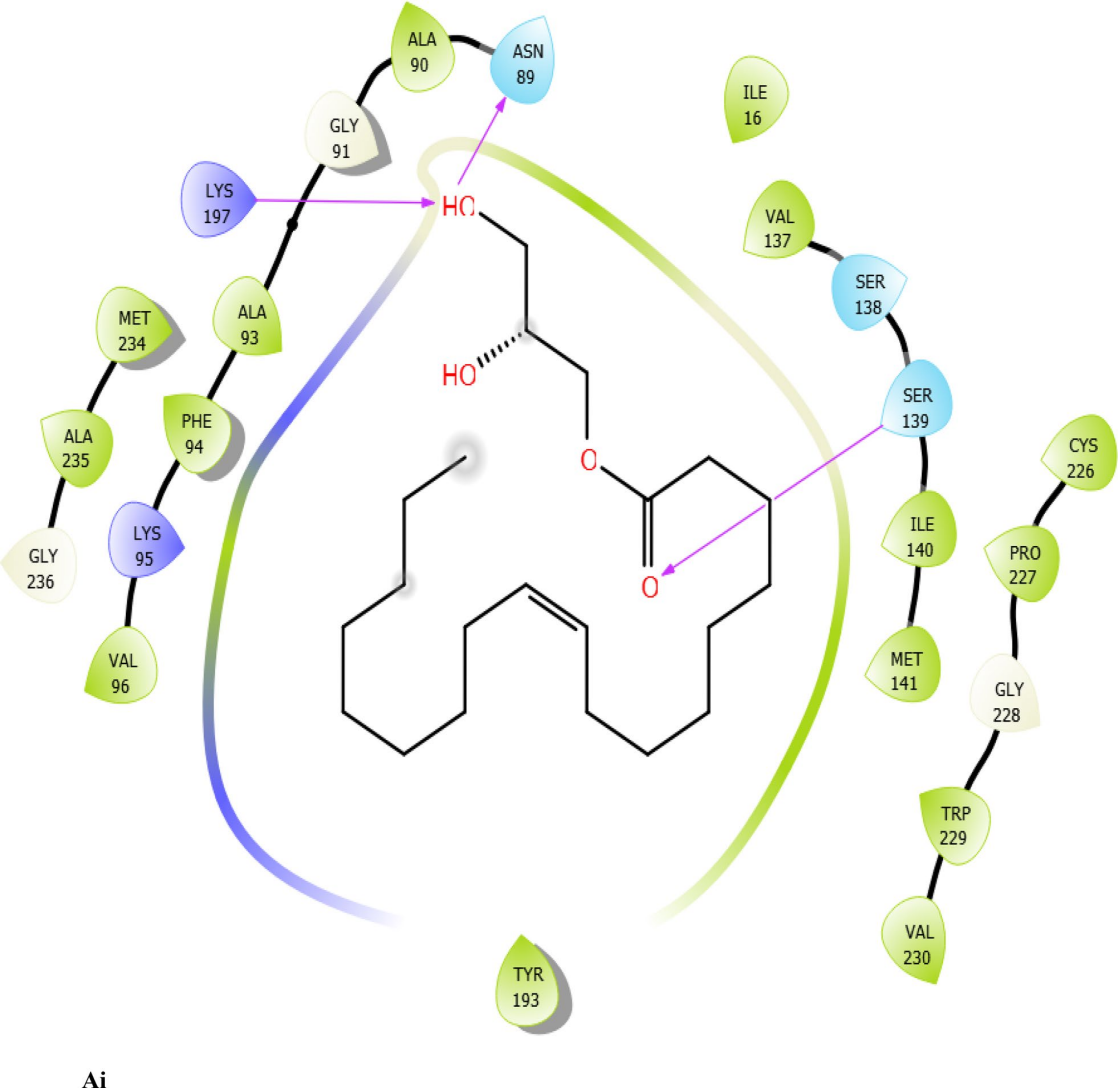

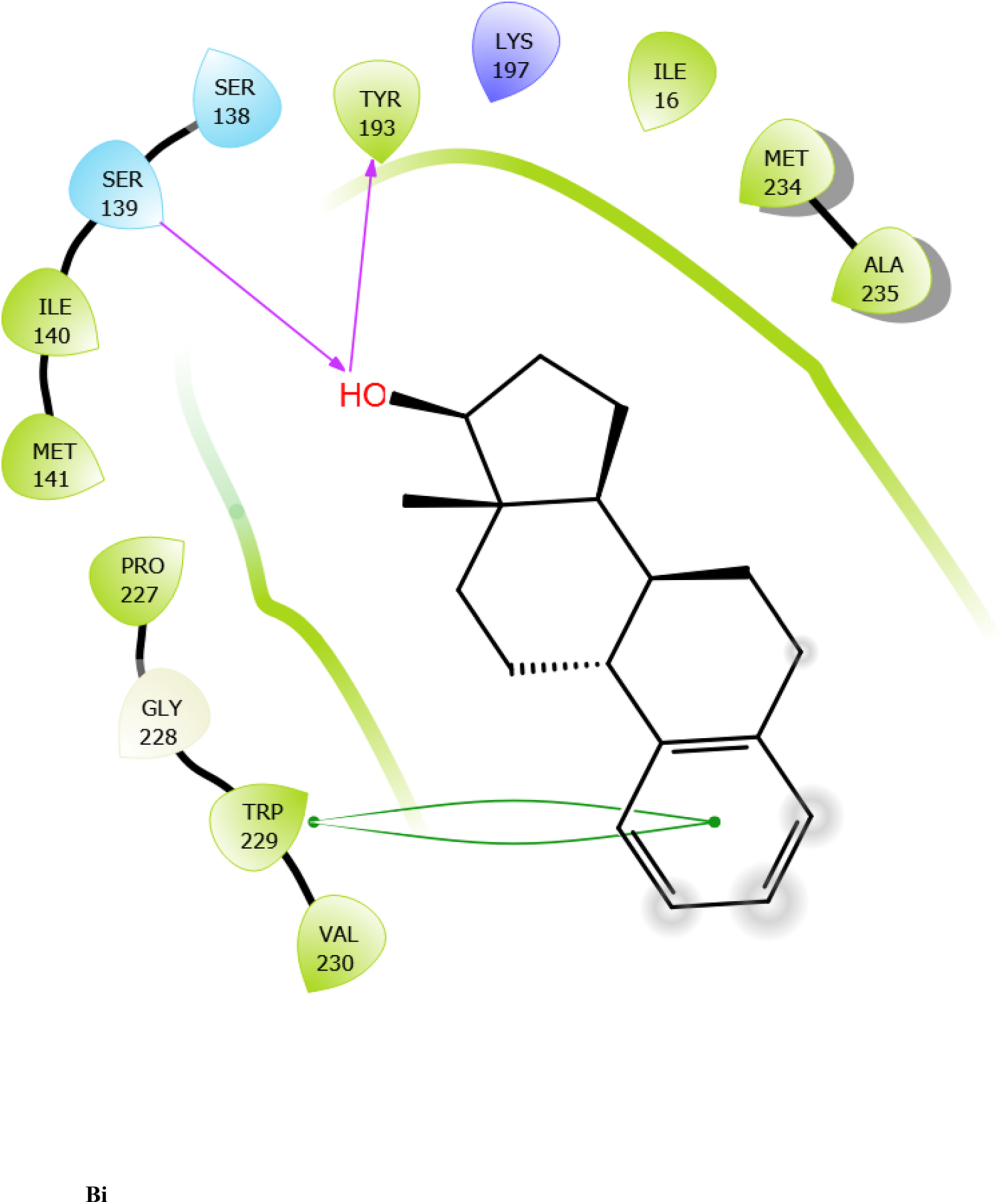

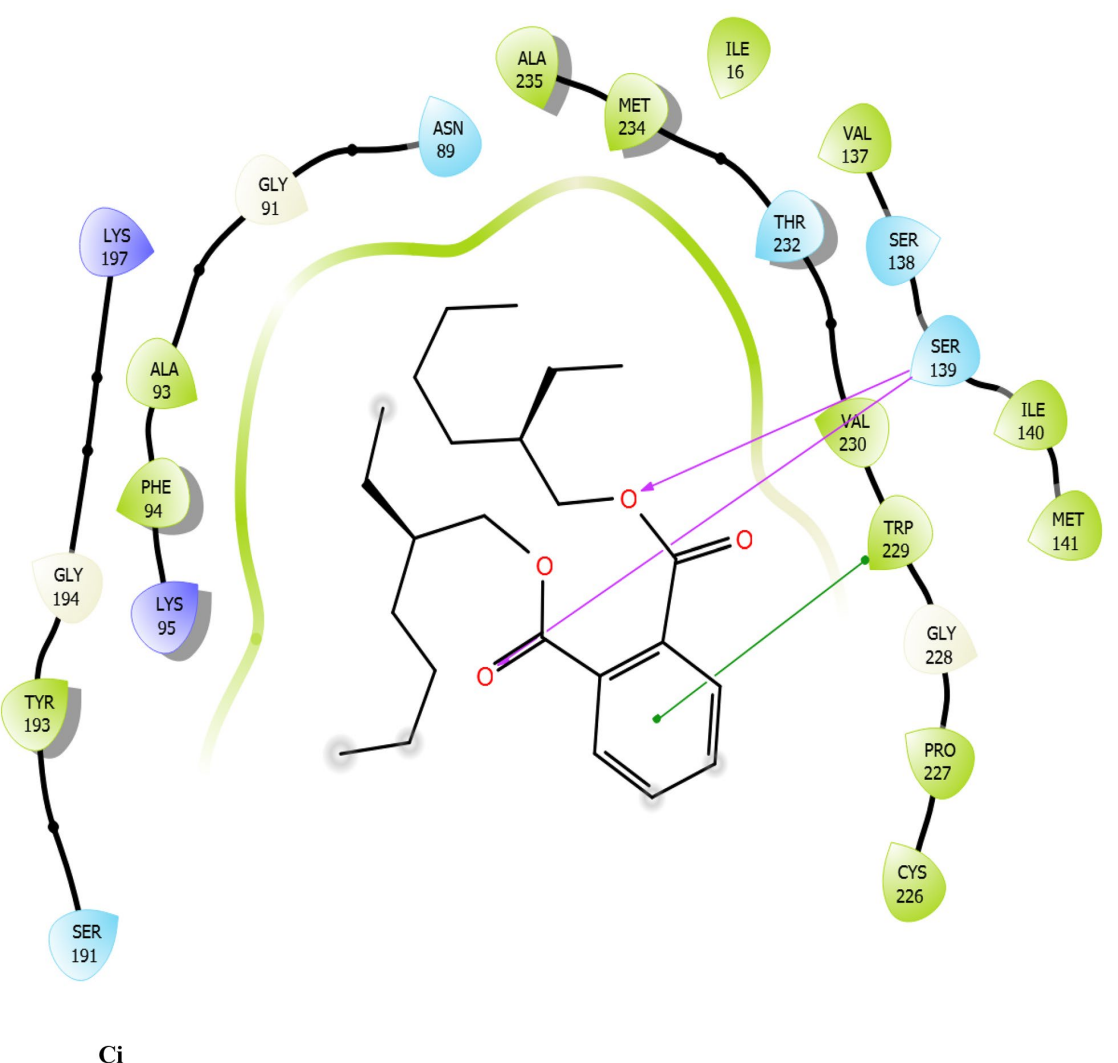

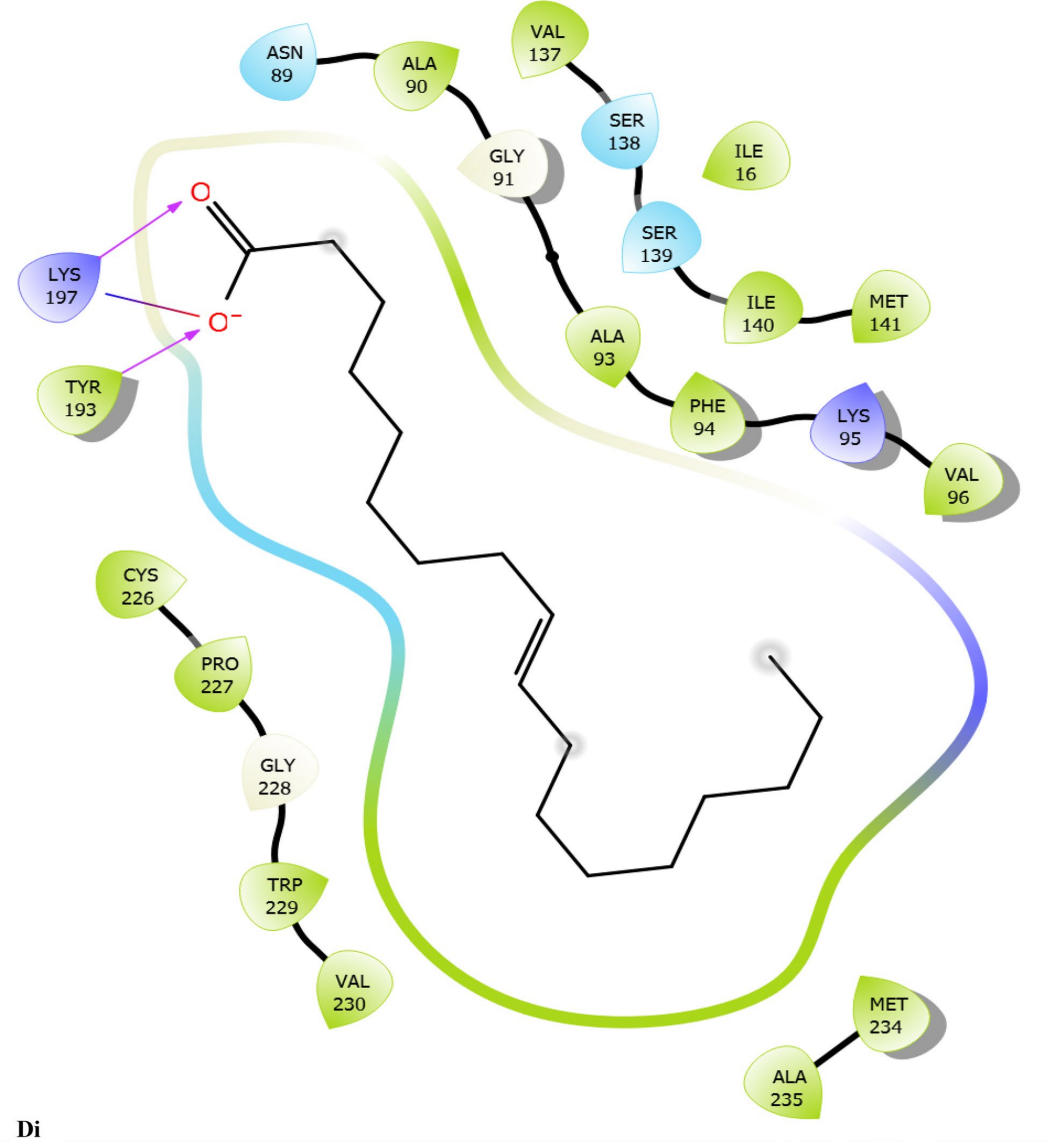

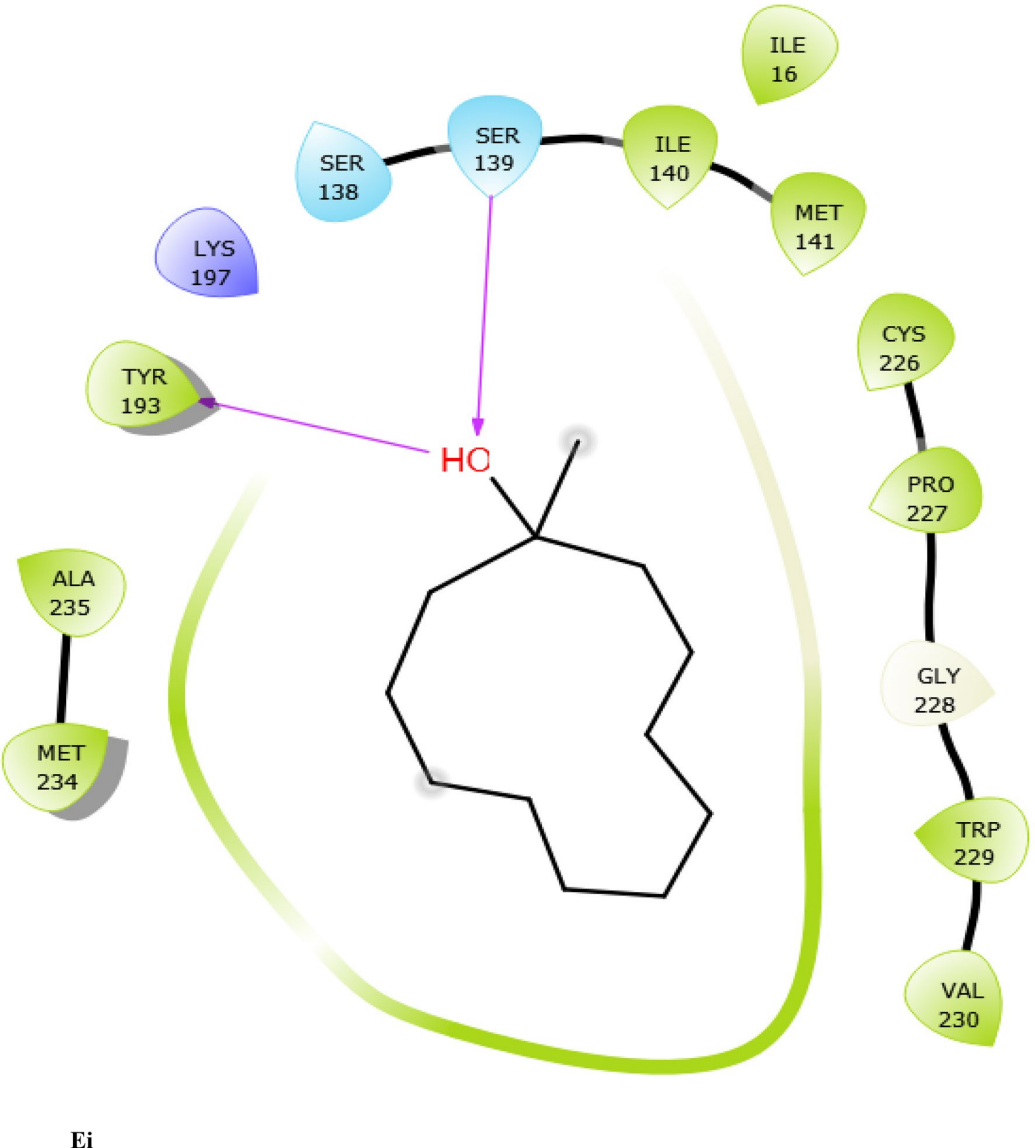

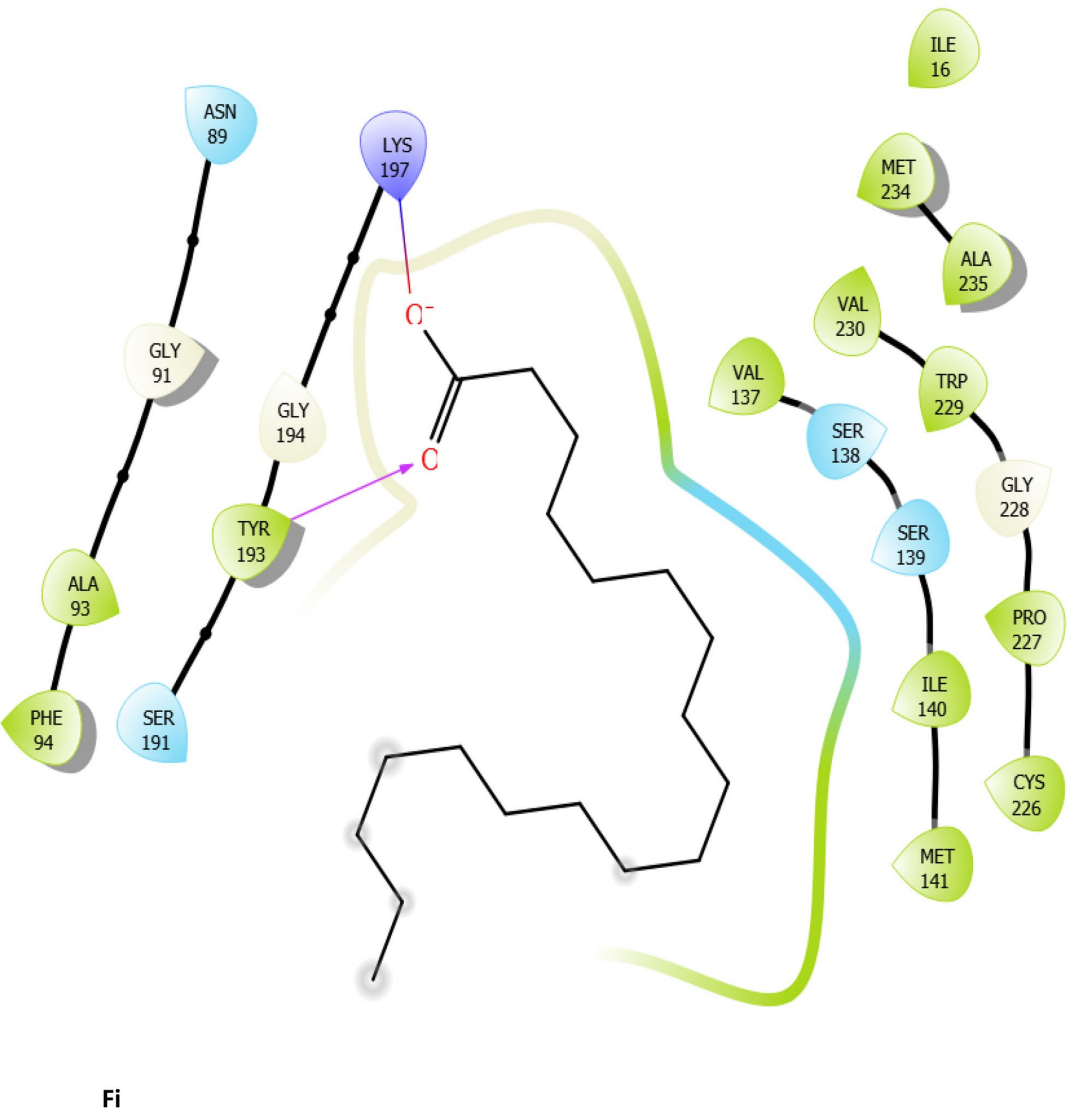

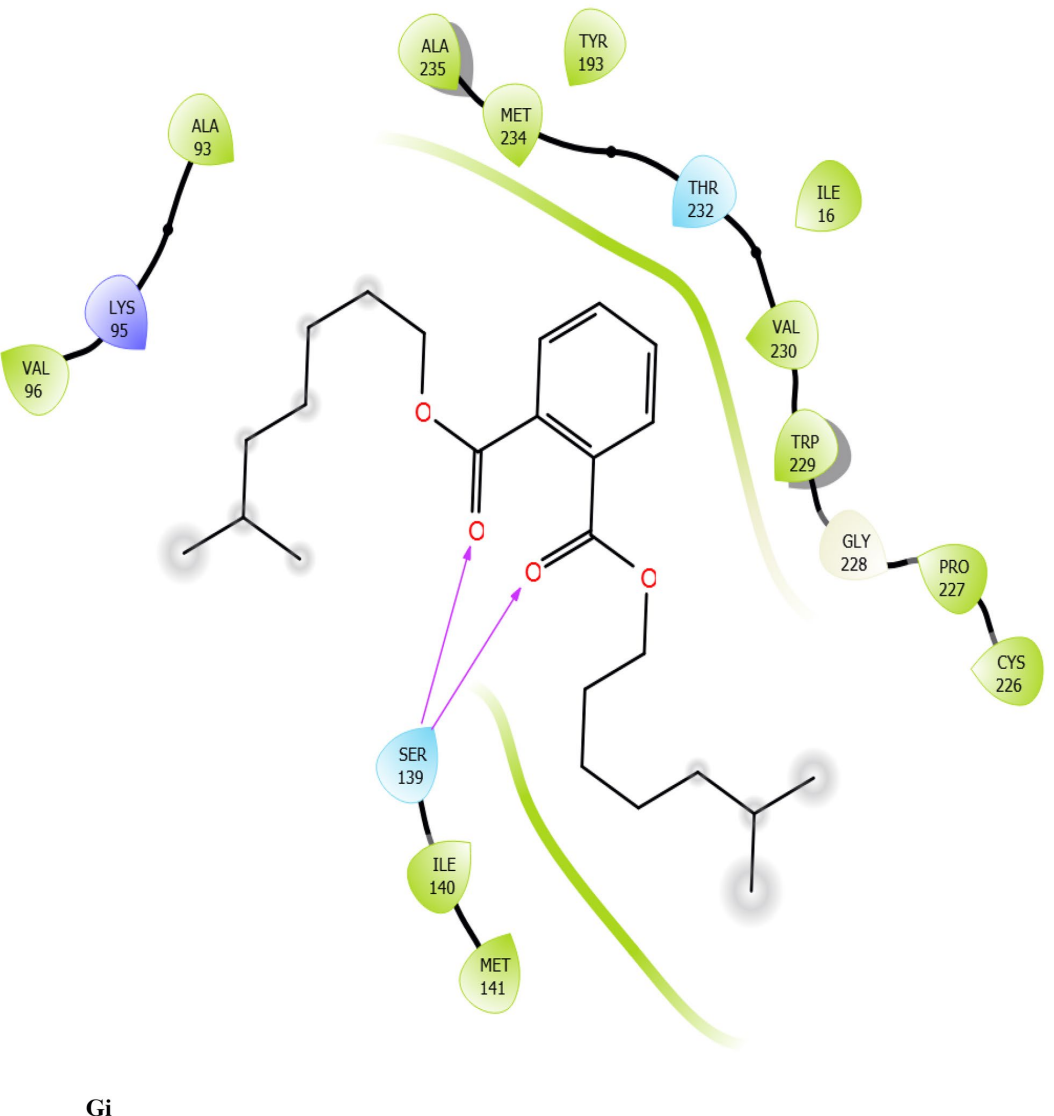

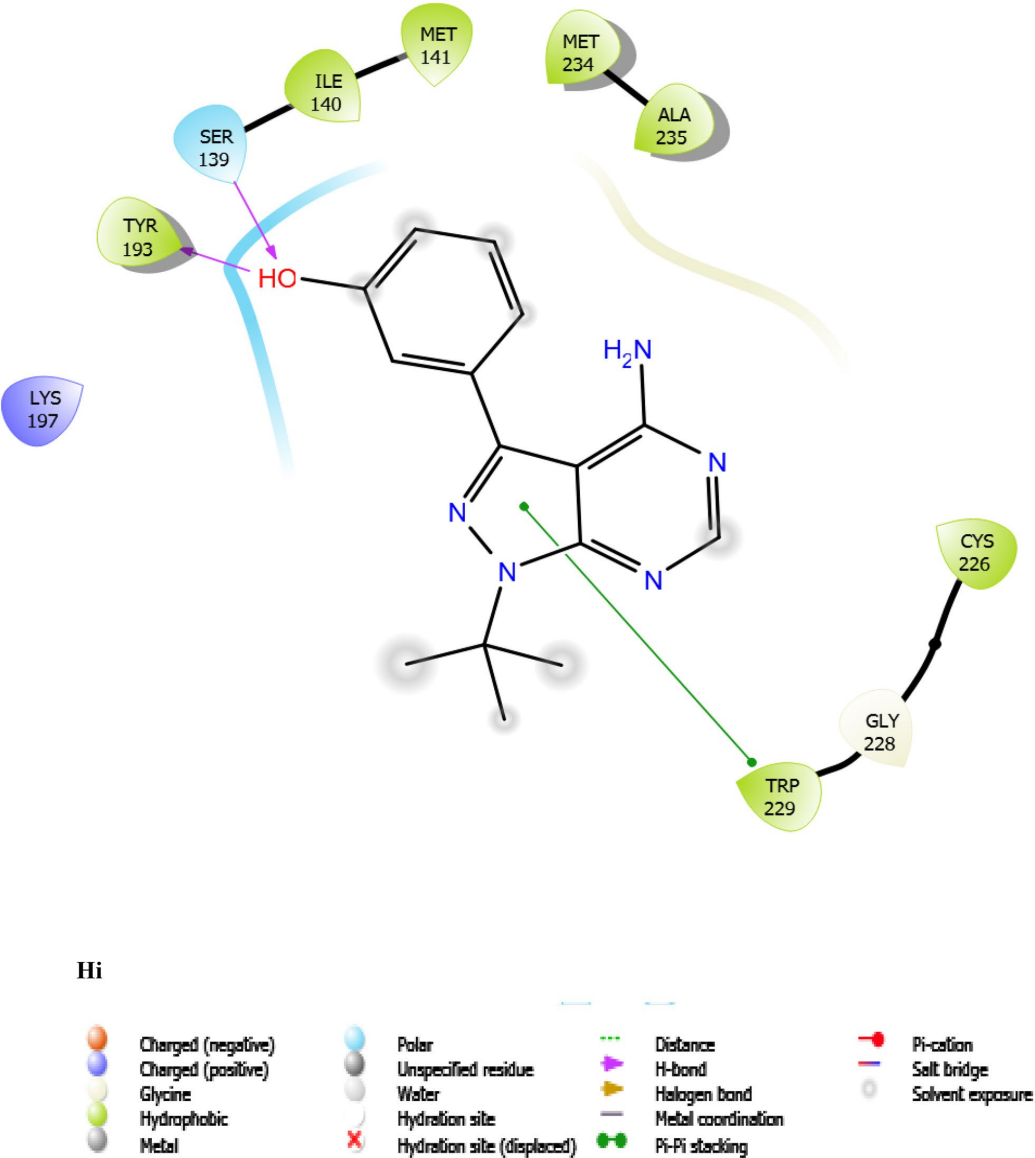
2D interaction of ligands with the target protein (1WMA). (Ai) 9-Octadecenoic acid (Z)-, 2,3-dihydroxy propyl ester. (Bi) Estra-1,3,5(10)-trien-17. beta. –ol. (Ci) Bis(2-ethylhexyl) phthalate (Di) 9-Octadecenoic acid. (Ei) Cycloundecanol, 1-methyl. (Fi) Octadecanoic acid. (Gi) Diisooctyl phthalate. (Hi) co-crystallized ligand

Section of the heart presented in the control group (Fig. 6a) showed the normal myocardial histomorphology for laboratory rodents. The sections displayed the normal epicardial, myocardial, and endocardial layers of the cardiac muscle. The myocardium showed normal elongated myocytes arranged in overlying bundles, enveloped by a rich mesh of blood vessels and capillaries embedded in a connective tissue matrix. The cardiac myocytes contain single centrally located oval to elongated hypochromatic nuclei (white arrow). Fibrocytes of the connective tissue matrix unfold as spindle shaped cells with spindle shaped hyperchromatic nuclei (black arrow). Capillaries (Red arrow). The DOX 20 mg/kg b.w. (Fig. 6b) showed section of the heart with multifocal regions of myocardial deterioration and necrosis with odema (arrow) and marked infiltration of inflammatory leukocytes. Blood vessel (BV). Normal myocardial histomorphology was observed in the section of heart presented in the Vasoprin 150 mg/kg b.w. (Fig. 6c). Nuclei (white arrow); pericytes (black arrow); cappilaries (red arrow). ERBECP 200 mg/kg b.w (Fig. 6d) and ERBECP 400 mg/kg b.w. (Fig. 6e) rats showed sections of the heart with moderate myocardial degeneration and odema (arrow) with mild infiltration of inflammatory leukocytes. ERBECP 600 mg/kg b.w. (Fig. 6f) treated with the highest dose of the extract showed sections of the heart with a relatively normal myocardial histomorphology. However, very mild evidence of myocardial degeneration can still be observed (arrow) (Fig. 7).

### Molecular docking

The results of XP docking scores of ERBECP constituents are shown in Table 4. The 69 compounds screened against the target protein (CBR1), we identified seven hits based on the docking score and interactions with the active site residues. The study showed that of the seven selected compounds, 9-Octadecenoic acid (Z)-, 2,3-dihydroxy propyl ester had the highest docking score (− 6.942 kcal/mol) and Diisooctyl phthalate had the lowest docking score (− 4.700 kcal/mol).

**Table 4:** Schrodinger XP docking scores of *C. patens* constituents

Compound	PubChem ID	Chemical formula	Binding affinity (kcal/mol)
9-Octadecenoic acid (Z)-, 2,3-dihydroxy propyl ester	71,751,310	C_21_H_40_O_4_	− 6.942
Estra-1,3,5(10)-trien-17. beta. –ol	537,293	C_18_H_24_O	− 5.487
Bis(2-ethylhexyl) phthalate	8343	C_24_H_38_O_4_	− 5.356
9-Octadecenoic acid, (E)	637,517	C_18_H_34_O_2_	− 4.698
Cycloundecanol, 1-methyl	551,293	C_12_H_24_O	− 4.469
Octadecanoic acid	5281	C_18_H_36_O_2_	− 4.905
Diisooctyl phthalate	33,934	C_24_H_38_O_4_	− 4.700
Co-crystallized ligand	657,095	C_15_H_17_N_5_O	− 5.242

Table 5 shows the drug-likeness prediction of ERBECP constituents. All the *C. patens* constituents had MW (g/mol) less than 500, HBA < 10 and HBD not more than 5. All the constituents demonstrated moderate water solubility except Bis(2-ethylhexyl) phthalate, Octadecanoic acid and Diisooctyl phthalate that are poorly soluble. Notably, the entire constituent violated either one or no Lipinski’s rule of five for orally administered drugs.

**Table 5:** Drug-likeness Prediction of *C. patens* constituents

Compounds	MW (g/mol)	No. HBA	No. HBD	cLog*P value*	GIA	No. violation	TPSA (Å)	Water solubility
9-Octadecenoic acid (Z)-, 2,3-dihydroxy propyl ester	356.54	4	2	5.07	High	No	66.76	Moderately soluble
Estra-1,3,5(10)-trien-17. beta. -ol	256.38	1	1	3.83	High	1	20.23	Moderately soluble
Bis(2-ethylhexyl) phthalate	390.56	4	0	6.17	High	1	52.60	Poorly soluble
9-Octadecenoic acid, (E)	286.46	2	1	5.71	High	1	37.30	Moderately soluble
Cycloundecanol, 1-methyl	184.32	1	1	3.34	High	No	20.23	Soluble
Octadecanoic acid	284.48	2	1	5.93	High	1	37.30	Poorly soluble
Diisooctyl phthalate	390.56	4	0	6.50	High	1	52.60	Poorly soluble

Table 6 shows the toxicity properties of ERBECP constituents. Apart, 9-Octadecenoic acid (Z)-, 2,3-dihydroxy propyl ester and Estra-1,3,5(10)-trien-17. beta. -ol, all the constituents had LD_50_ lower than 2000 mg/kg. The toxicity prediction showed relative safety of ERBECP constituents except for Estra-1,3,5(10)-trien-17. beta. -ol, Octadecanoic acid and Diisooctyl phthalate which showed potentials for immuno-toxicity and carcinogenicity.

**Table 6:** Toxicity Properties of *C. patens* constituents

Compounds	Predicted LD_50_ value (mg/kg)	Prediction accuracy (%)	Hepato-toxicity	Immuno-toxicity	Cytotoxicity	Carcinogenicity	Mutagenicity
9-Octadecenoic acid (Z)-, 2,3-dihydroxy propyl ester	39,800	70.97	Inactive	Inactive	Inactive	Inactive	Inactive
Estra-1,3,5(10)-trien-17. beta. –ol	5010	69.26	Inactive	Active	Inactive	Inactive	Inactive
Bis(2-ethylhexyl) phthalate	1340	100	Inactive	Inactive	Inactive	active	Inactive
9-Octadecenoic acid, (E)	48	100%	Inactive	Inactive	Inactive	Inactive	Inactive
Cycloundecanol, 1-methyl	1000	100%	Inctive	Inactive	Inactive	Inactive	Inactive
Octadecanoic acid	900	100%	Inactive	Active	Inactive	Inactive	Inactive
Diisooctyl phthalate	1340	100	Inactive	Inactive	Inactive	Active	Inactive

## Discussion

Despite decades of good results in the clinical application of DOX in cancer therapy, this drug induces cumulative, dose-dependent toxicity and adverse effects, such as cardiotoxicity, and affects the kidney and liver [30]. The inhibition of drug efflux transporters p-glycoprotein and cancer-resistant protein restores the intracellular levels of drug in DOX-resistant and leads to the retention of DOX [31]. Our research suggests that the investigated extract could inhibit one or more of these proteins and induce the retention of DOX. As the formation of free radicals is considered as the major culprit in DOX-induced cardiotoxicity, antioxidant compounds found in this plant could be recognized as potential therapeutic agents.

Free radical formation which culminates in oxidative damage to the cardiac muscle remains the most accepted hypothesis suggested for doxorubicin-induced myocardial infarction [32–34]. According to literature, several plant derived phytochemicals elicit cardioprotective effects in vitro and in vivo against DOX-triggerred cardiotoxicity [35, 36]. These phytochemicals represent natural reservoirs of pharmacophore that act as templates for developing safe and promising novel cardioprotective agents [36]. This study investigated the cardioprotective potential of the ERBECP in DOX-induced myocardial infarcted rats. The results of the phytochemical analysis revealed the abundant presence of terpenoids, phenols, flavonoids and alkaloids which are known to be rich in antioxidant activity [37–39].

The GC–MS analysis of ERBECP identified a total of 69 compounds, these compounds properties in ERBECP could be attributed to the presence of these essential phytoconstituents with antioxidant potentials. The acute toxicity test showed that ERBECP was not lethal even after ERBECP administration at the highest dosage 5000 mg/kg b.w. showing that the plant is safe for both human and animal consumption. The changes of b.w. of rats in different groups were monitored for. The results showed a significant (*p* < 0.05) increases in final b.w. of rats in comparison to the values obtained for the initial b.w. rats. Pre-treatment of DOX group with ERBECP could significantly increase the body weight compared to the DOX-treated group during the experimental period of 2 weeks. Several reports have revealed the direct toxic effects of chemotherapy drugs, such as doxorubicin, on the gastrointestinal tract including the toxic effects on intestinal mucosa which appear as mucositis, leads to reduce food intake and weight loss [40–43].

CTnI, CK, LDH, AST and ALT were estimated as cardiac biomarker enzymes. In the present study, DOX control group (DOX 20 mg/kg b.w) showed a marked increase in level of these cardiac biomarker enzymes indicating that there is an extensive damage to the cardiac tissues in the presence of DOX. DOX has the potential to disrupt the cell membrane, enabling the leakage of intracellular proteins such as CTnI and CK which are absolutely peculiar to cardiac function [44] whereas the enhanced levels of LDH, AST and ALT in the serum are not ideal diagnostic parameters because they could depict non-cardiac conditions such as hepatic diseases [45–47]. Cardiac troponin is found the cardiac muscle and acts on muscle contraction by regulating the calcium-dependent interaction of actin and myosin [48, 49]. The increased level of CTnI is a highly sensitive and specific index of myocardial necrosis in humans and animals [50]. CK reversibly phosphorylates creatine and remains a valuable index of myocardium damage [48, 51]. Pre-reatment with the ERBECP 200, 200 and 600 mg/kg b.w and as well Vasoprin 150 mg/kg b.w substantially reduced the levels of these biomarker enzymes in circulation and might be due to its effect in maintaining membrane integrity. This result harmonizes with those of [9, 50, 52], demonstrating that DOX caused oxidative damage to heart tissue via lipid peroxidation and that plant extracts with antioxidant properties are protective against DOX-induced myocardial insult.

The previous studies by [53–55] reported plants extract to reduce cardiac cell injury caused by DOX. The antioxidant mechanism of ERBECP may include one or more of the following interactions; scavenging or neutralizing of free radicals[56], inhibition of oxidative enzymes like cytochrome P_450_ [57], oxygen quenching and making it less available for oxidative reaction, interacting with oxidative cascade and preventing its outcome [58], and disarming oxidative properties of metal ions such as iron [59]. Thus, in this work, ERBECP effectively prevented tissue damage by decreasing the oxidative stress and restoring the antioxidant status. Myocardial infarction is also associated with dyslipidemia [60]. In this context, the DOX 20 mg/kg b.w exhibited a remarkable increase in TC, TAG, LDL and decrease in HDL. However, administration of ERBECP 200, 200 and 600 mg/kg b.w and as well Vasoprin 150 mg/kg b.w normalized these alterations of the lipid profile parameters. This is consistent with the findings of [18, 61], and also that of [62] in isoproterenol-induced myocardial infarction. Polyphenols have been reported to inhibit cholesterol esterase which plays a significant role in liberating free cholesterol from the small intestine by hydrolyzing dietary cholesterol esters [62]. The increased concentration of total cholesterol could be as a result of a decrease in HDL [63]. HDL functions in the transport of cholesterol from tissues to the liver for catabolic degradation and is believed to be antiatherogenic and antagonistic to the pathways of inflammation, thrombosis and oxidation [60]. Also, the inactivity of lipoprotein lipase may be responsible for the increased level of TAG in circulation [63]. Free radicals generated by DOX promote LDL oxidation and oxidized LDL is responsible for the development of atherosclerosis [63]. HDL limits LDL oxidation thereby preventing myocardial damage [64].

Lipid peroxidation is an integral process for free radical mediated cell injury and an oxidative transformation of polyunsaturated fatty acids (PUFAs) to end products such as MDA [65, 66]. Increase in MDA concentration in the DOX 20 mg/kg b.w illustrates augmented lipid peroxidation which may be attributed to an inadequate antioxidant protection process. This agrees with sudies conducted by [67, 68]. SOD catalyzes the conversion of superoxide radicals to hydrogen peroxide which is ultimately converted to water and molecular oxygen by CAT [69, 70]. GSH is a non-enzymatic antioxidant and a tripeptide of glutamate, cysteine and glycine [65]. GSH reduces toxic peroxides and maintains the active state of enzymes by impeding the conversion of sulfhydryl group to disulfide group [71]. The dimunition of the catalytic activities of SOD, CAT and GSH levels in the DOX 20 mg/kg b.w agrees with the findings of [69, 72]. Pre-treatment with the ERBECP 200, 200 and 600 mg/kg b.w and as well Vasoprin 150 mg/kg b.w greatly improved the activities of these endogenous antioxidants and simultaneously reduced MDA levels. The extract rich in antioxidant bioactive constituents may have deployed free radical scavenging mechanisms for instance flavonoids stabilize reactive oxygen species and due to the high spontaneity of their hydroxyl moiety, radicals are inactivated [38]. They may also have a supplemental effect to the endogenous antioxidative compounds in the cardiac muscle cells [38, 73].

Histopathological assessment of the heart muscle tissue of different groups further reinforced the findings of biochemical assessment. The control group (a) represented evident integrity of the cardiomyocyte membrane and no inflammatory cell invasion was observation. The DOX 20 mg/kg b.w group (b) exposed section of the heart with portions of myocardial necrosis, odema and marked infiltration of inflammatory leukocytes. Pre-treatment with ERBECP 200, 200 and 600 mg/kg b.w and standard drug Vasoprin 150 mg/kg b.w considerably counteracted the pathological effects of DOX as noted in the reduced inflammatory cell invasion and relatively normal view of myocardial cells. This observed protective function may be attributed to the different or synergistic effect of bioactive phytocompounds present in the crude extract.

To evaluate the binding interactions of ERBECP constituents, a molecular docking study was performed at the active site of carbonyl reductase (CBR1) active site. This gives a better understanding of the in vitro experimental results. Doxorubicin is metabolized by a class of cytosolic enzymes called carbonyl reductases. When doxorubicin dosage is sufficiently high, carbonyl reductases convert DOX to doxorubicinol. This alcohol metabolite builds up in cardiac tissues, impairing both systolic and diastolic cardiac function [25]. Studies have recommended inhibitors of CBR1 as an adjunct to Doxorubicin therapy to ameliorate cardiac side effects [25]. Observations from the molecular docking results showed that ERBECP constituents had overall binding energies in ranges of − 6.9 to 4.7 kcal/mol. 9-Octadecenoic acid (Z)-, 2,3-dihydroxy propyl ester, Estra-1,3,5(10)-trien-17. beta. -ol and Bis(2-ethylhexyl) phthalate exhibited a higher binding affinity when compared with the cognate crystallized ligand. However, the cognate crystallized ligand had a higher binding affinity when compared to Cycloundecanol, 1-methyl, Octadecanoic acid, Diisooctyl phthalate, 9-Octadecenoic acid, (E)-. This may be due to the presence of a bulkier methyl group resulting in fewer hydrogen bond interactions.

The interaction between active site residues is a direct consequence of the orientations of these ligands [74]. The substrate-binding site of CBR1 is surrounded mainly by hydrophobic residues such as Trp229, Met141 and Ile140 [75]. Deep into the substrate binding site are Ser 139 and Tyr193 of the catalytic triad. The interaction of these residues with the ligands has been shown to play a crucial role in the inhibition of the protein [76]. Visual observation of the docked conformation showed that Estra-1,3,5(10)-trien-17. beta. -ol interacted with the core residues at the substrate binding site of the target. The hydroxyl group at position 17 of the steroid nucleus (D ring) of Estra-1,3,5(10)-trien-17. beta. -ol was engaged in a hydrogen bond interaction with Ser 139 and Tyr193. The co-crystallized ligand and Cycloundecanol, 1-methyl- also demonstrated a similar interaction. This may suggest that the hydroxyl moiety is a crucial interaction determinant with the catalytic machinery of CBR1, indicating that the presence of this functional group is strongly required for CBR1 inhibition [62]. Estra-1,3,5(10)-trien-17. beta. -ol and Bis(2-ethylhexyl) phthalate also showed pi-pi stacking interaction with Trp 229 respectively.

Regardless of a drug’s oral activity, Lipinski's rule of five can be utilized to determine whether a pharmacologically active molecule possesses drug-like qualities [77]. According to Lipinski’s rule of five, the logP value which is a measure of the lipophilicity of a compound must be less than five. HBD and HBA must be less than five and ten respectively [78]. Additionally, the molecular weight must be less than 500 g/mol and the Topological polar surface area must be less than 140 Å [74]. Only one of these guidelines must be broken by molecules for them to be accepted. As shown in Table VIII, 9-Octadecenoic acid (Z)-, 2,3-dihydroxy propyl ester, and Cycloundecanol, 1-methyl- did not violate any of the Lipinski’s guidelines. However, Estra-1,3,5(10)-trien-17. beta. -ol, Bis(2-ethylhexyl) phthalate, Octadecanoic acid, Diisooctyl phthalate and 9-Octadecenoic acid violate only one of the guidelines. All the compounds showed high GIA absorption suggesting the compound has increased bioavailability. However, Estra-1,3,5(10)-trien-17. beta. -ol and Octadecanoic acid demonstrated immunotoxicity. While Diisooctyl phthalate showed carcinogenicity. Further in vitro and in vivo toxicity studies are however needed to determine the efficiency and safety of these compounds.

## Conclusions

The biochemical findings and molecular docking obtained from this study indicate that the ERBECP offers protection to the myocardium against DOX-induced myocardial infarction in rats. The resultant cardioprotective effect could be due to the augmentation of the antioxidant defense network. Hence, the ERBECP can be subjected to further research to enable the isolation of lead compounds useful in the development of adjuvants that can be used in combination with DOX to possibly combat its toxic effects and also to synthesize novel therapeutic agents for ameliorating cardiac health burdens.

## Data Availability

The datasets used and/or analysed during the current study are available from Chidinma Pamela Ononiwu or the corresponding author on reasonable request.

## Abbreviations

ALT: Alanine aminotransferase
ANOVA: One-way analysis of variance
AST: Aspartate aminotransferase
CAT: Catalase
CK: Creatine kinase
CTnI: Cardiac troponin-I
DOX: Doxorubicin
ECG: Electrocardiograhic
ELISA: Enzyme linked-immunosorbent assay
ERBECP: Ethanol root bark extract of *Cleistopholis Patens*
GC–MS: Chromatography-mass spectometry
GSH: Glutathione
HDL: High density lipoprotein
InterCEDD: International center for economic and drug development
LDH: Lactate dehydrogenase
LDL: Low density lipoprotein
MDA: Malondialdehyde
NADPH: Nicotinamide adenine dinucleotide phosphate
NOS: Nitric oxide synthases
OPLS4: Optimal potentials for liquid simulations
RMSD: Root-mean-square deviation
ROS: Reactive oxygen species
SOD: Superoxide dismutase
SPSS: Statistical product and service solution
TAG: Triacylglycerol
TC: Total cholesterol
